# Physiognomy of the Nose

**Published:** 1887-07

**Authors:** Joseph Simms

**Affiliations:** The Distinguished Physiognomist


### PHYSIOGNOMY OF THE NOSE.

By Dr. Joseph Simms the Distinguished Physiognomist,

(Copyright by J. Simms M. D. 1887.)

As a general principle, large noses are indicative of active, energetic characters, apt to be proud, pompous, impatient, desirous of being leaders and commanders, and often overbearing and tyrannical. On the contrary, small low noses denote weak characters, deficient in government, even of themselves and slaves of their appetites, loves and hates, rather than persons guided by reason and judgment. The large-nosed, in critical positions and circumstances of excitement will be cool and self-possessed, and competent to act more prudently than the small-nosed. Large noses are found chiefly among the inhabitants of mountainous regions and their descendants; small ones originate in low, flat countries. When the nose is long in proportion to its general size, it bears the impress of discretion, timidity, caution aud thoughtfulness. Noses relatively short from the forehead to the point evince rashness, carelessness and self-will; while noses that stand out prominently represent characters that are discontented with their present lot and are anxious and aspiring. But when the point of the nose clings to the upper lip the tendency is to be miserly and to love earthly things. The nose that is thin as well as generally small, prefigures a poor, weak constitution and feeble character, with a tendency to consumption, presaging an early death. On the other hand if the nose is thick where it joins the face we feel assured of a strong constitution and strong passions, and have good reason to expect long life if proper care and prudence be exercised. Persons with sharp pointed noses are keen, intense, penetrating, and mostly quick tempered. A nose thick and nearly square at the point denotes a talent for invention and an earnest desire to progress and excel. A nose that is prominent and almost straight, seeming to have two points formed by a vertical depression through the end signalizes a logical and meditative mind. A person whose nose reaches toward the mouth is cautious, but is specially considerate about bodily wants. Noses projecting in a straight line forward at the base imply that their possessors are of quiet dispositions, regular habits, especially in middle life. Round, knobby noses are mostly connotative of speculative minds, retentive memory and musical tastes. The small low round nose generally known as the pug, turning up a little at the point, signifies a pert, forward, saucy, conceited individual. A nose that shows a large proportion of bone in its size denotes a stable character, slow deliberate judgment, firm and reliable ; while the soft fleshy or gristly nose is expressive of a sly deceptive, cunning, treacherous character. The bony nose originates generally in temperate climates, and the gristly fleshy, in the torrid zone. Examples of the gristly nose may be seen in the cat and all other members of the feline species. The straight nose inclines to science, art, polite literature and political economy if duly educated thereto. But the nose of convex form from the forehead to the point is emblematic of the inclination, for commercial pursuits and true speculative talent. A dull obtuse intellect with much physical power and destructive inclination, is typified by a nose very broad at the base. When the lower portion of the nose forms an obtuse angle with the face, and the point is elevated about 45 degrees, we see a person inclined to snobbery and fashion. If the septum is longer than the sides we may infer an original and suggestive mind, as well as a penetrating and sagacious one. The nose that is high and thin in the upper part bespeaks moral courage, love of argument, quick apprehension, capacity to use to the greatest advantage what is known or at hand. Wide spread nostrils argue strong lungs, while the closing of them betokens pulmonary weakness. Wrinkles across the top of the nose are signs of thoroughness in every sense. A fiery, red, warty and enlarged nose, betrays a dimunition of energy through disease or strong drink, or scrofulous inherited tedencies, or excessive study while living principally on animal food, without sufficient vegetable diet. Long, sharp, and well formed noses have an acute scent, if not subject to catarrh. As a general rule, square noses symbolize a masculine, and round ones a feminine character. The lower animals, as well as human beings with long noses, are uneasy, watchful, suspicious and prone to travel. Those with short noses, are slow of movement. If the bridge of the nose is high, it evinces a disposition to assail those that are considered to be doing wrong.

---

The foregoing interesting and instructive article, is copyrighted, but we are authorized to give permission to other publications to copy it, provided they give credit to its author and to this Journal and mention the fact of the copyright —[Ed.]

